# TGF-β mRNA levels in circulating extracellular vesicles are associated with response to anti-PD1 treatment in metastatic melanoma

**DOI:** 10.3389/fmolb.2024.1288677

**Published:** 2024-04-03

**Authors:** Stefania Crucitta, Federico Cucchiara, Riccardo Marconcini, Alessandra Bulleri, Simona Manacorda, Annalisa Capuano, Dania Cioni, Amedeo Nuzzo, Evert de Jonge, Ron H. J. Mathjissen, Emanuele Neri, Ron H. N. van Schaik, Stefano Fogli, Romano Danesi, Marzia Del Re

**Affiliations:** ^1^ Unit of Clinical Pharmacology and Pharmacogenetics, Department of Clinical and Experimental Medicine, University of Pisa, Pisa, Italy; ^2^ Unit of Medical Oncology 2, Department of Medicine and Oncology, Azienda Ospedaliero-Universitaria Pisana, Pisa, Italy; ^3^ Unit of Radiodiagnostics 1, Department of Translational Research and New Technologies in Medicine and Surgery, University of Pisa, Pisa, Italy; ^4^ Campania Regional Centre for Pharmacovigilance and Pharmacoepidemiology, Section of Pharmacology, Department of Experimental Medicine, University of Campania “Luigi Vanvitelli”, Napoli, Italy; ^5^ Department of Clinical Chemistry, Erasmus University Medical Center, Rotterdam, Netherlands; ^6^ Department of Medical Oncology, Erasmus University Medical Center Cancer Institute, Rotterdam, Netherlands; ^7^ Department of Oncology and Hemato-Oncology, University of Milano, Milano, Italy

**Keywords:** TGFβ, immunotherapy, melanoma, extracellular vesicles, liquid biopsy

## Abstract

**Introduction:** Immune checkpoint inhibitors (ICIs) represent the standard therapy for metastatic melanoma. However, a few patients do not respond to ICIs and reliable predictive biomarkers are needed.

**Methods:** This pilot study investigates the association between mRNA levels of programmed cell death-1 (PD-1) ligand 1 (PD-L1), interferon-gamma (IFN-γ), and transforming growth factor-β (TGF-β) in circulating extracellular vesicles (EVs) and survival in 30 patients with metastatic melanoma treated with first line anti-PD-1 antibodies. Blood samples were collected at baseline and RNA extracted from EVs; the RNA levels of PD-L1, IFN-γ, and TGF-β were analysed by digital droplet PCR (ddPCR). A biomarker-radiomic correlation analysis was performed in a subset of patients.

**Results:** Patients with high TGF-β expression (cut-off fractional abundance [FA] >0.19) at baseline had longer median progression-free survival (8.4 vs. 1.8 months; *p* = 0.006) and overall survival (17.9 vs. 2.63 months; *p* = 0.0009). Moreover, radiomic analysis demonstrated that patients with high TGF-β expression at baseline had smaller lesions (2.41 ± 3.27 mL vs. 42.79 ± 101.08 mL, *p* < 0.001) and higher dissimilarity (12.01 ± 28.23 vs. 5.65 ± 8.4; *p* = 0.018).

**Discussion:** These results provide evidence that high TGF-β expression in EVs is associated with a better response to immunotherapy. Further investigation on a larger patient population is needed to validate the predictive power of this potential biomarker of response to ICIs.

## 1 Introduction

Immune checkpoint inhibitors (ICIs) significantly increase survival in patients with melanoma and are the standard of therapy in the metastatic setting (Robert et al., 2015a; Robert et al., 2015b; Weber et al., 2015). In particular, the anti-PD-1 antibodies pembrolizumab and nivolumab induce objective response rates in 40%–45% of patients with advanced unresectable melanoma in the first-line setting (Robert et al., 2015a; Robert et al., 2015b; Larkin et al., 2015; Weber et al., 2015; Hamid et al., 2019). However, drug resistance may be observed since the beginning of treatment and some patients, who display initial response, may develop disease progression shortly thereafter (Andrews and Wargo, 2017; Zaretsky et al., 2016; ODonnell et al., 2017; Sade-Feldman et al., 2017). Unfortunately, reliable predictive biomarkers of response to ICIs in melanoma are lacking (Carlino et al., 2021). Therefore, the identification of potential biomarkers with predictive value represents a priority to optimize the treatment of melanoma with ICIs (Gibney et al., 2016; Hurkmans et al., 2020a; Hurkmans et al., 2020b; de With et al., 2021).

After priming and peripheral activation, T cells infiltrate the tumor, thus inducing immune-mediated cell death. The T-cell inflamed phenotype is also characterized by the local production of cytokines, activation of interferon-γ (IFN-γ, Schroder et al., 2004) signaling, and upregulation of PD-L1, which induces immune tolerance (Gibney et al., 2016). Research efforts have been focused on the role of tumor-associated PD-L1 expression, particularly on transforming growth factor-β (TGF-β), which has attracted much attention as a biomarker because of its pleiotropic role (Bu et al., 2022). Melanoma cells produce TGF-β to drive disease progression, inhibit immune responses and provide an optimal microenvironment that facilitates tumor growth. Furthermore, TGF-β performs its tumor-promoting functions through direct effects on the motility and invasiveness of tumour cells and indirectly by modulating tumor stroma and extracellular matrix, promoting angiogenesis and inhibiting immune surveillance (Batlle and Massague, 2019; Bu et al., 2022).

Liquid biopsy may be used to study dynamic changes in the tumor after treatment with anticancer drugs because of its minimally invasive nature and ability to collect tumor nucleic acids released in the circulation. In recent years, extracellular vesicles (EVs) emerged as a novel approach of biomarker analysis in liquid biopsy applications, due to their easy traceability, high concentrations in body fluids and capability to prevent molecular cargo from degradation (Urabe et al., 2020; Goricar et al., 2021).

EVs are heterogeneous bodies constituted by a cell membrane-like lipid bilayer (Yanez-Mo et al., 2015) with a diameter ranging from 100 to more than 200 nm (Yanez-Mo et al., 2015; Thery et al., 2018). EVs are released by budding of endosomal or plasma membrane in the circulation and carry DNA, RNAs, proteins and lipids derived from parental cells (Kao and Papoutsakis, 2019; Teng and Fussenegger, 2020).

EVs have multiple functions, including cell-cell signaling (Kao and Papoutsakis, 2019), promoting tumor proliferation and progression (Hoshino et al., 2015; Konig et al., 2017; Vinik et al., 2020) and participating in the tumor immune escape by releasing proapoptotic molecules (Huber et al., 2005). Recent studies have highlighted the utility of EVs as promising multicomponent biomarker vehicles, which enable concomitant analyses of several molecules, with predictive and diagnostic value (Thery et al., 2018; Vasconcelos et al., 2019; Urabe et al., 2020; Goricar et al., 2021). Among such biomarkers, TGF-β has been also featured in cancer EV biology (Rodrigues-Junior et al., 2022).

Therefore, the present study evaluates the possible association between response to ICIs and mRNA levels of TGF-β, IFN-γ and PD-L1 in circulating EVs isolated from peripheral blood of patients with metastatic melanoma treated with first-line nivolumab or pembrolizumab; radiomic features are also investigated in a subset of patients.

## 2 Materials and methods

### 2.1 Patients and data collection

In this study we included patients older than 18 years of age, affected by histologically confirmed metastatic melanoma, candidates to start a first-line treatment with anti-PD1, naïve for previous treatments. Patients were given nivolumab (240 mg i.v. every 2 weeks) or pembrolizumab (200 mg i.v. every 3 weeks), both as monotherapy, as part of their routine clinical management. Complete (CR) or partial responses (PR), disease stabilization (SD), and disease progression (PD) were defined by RECIST (v. 1.1) criteria (Eisenhauer et al., 2009; Hodi et al., 2016). This single cohort prospective study was approved by the Ethics Committee of Pisa University Hospital (acronym Exobiox, prot. n. 12159 of 8 June 2020) and conducted following the principles of the Declaration of Helsinki. All patients gave their signed informed consent before blood collection and data analysis.

### 2.2 Blood sampling and mRNA isolation from extracellular vesicles

Two blood samples (6 mL each) were obtained from patients at baseline for the assessment of PD-L1, INF-γ, and TGF-β mRNA levels in EVs. Blood samples were collected in EDTA tubes and centrifuged for 10 min at 1,900 g within 2 h; RNA contained in plasma-derived EV was extracted by SoRTEV™ RNA low volume kit (EXOSOMICS, Siena, Italy) starting from 2 mL of plasma samples. The isolation is based on immuno-affinity binding to beads coated with supplier’s proprietary antibodies against exosome surface antigens derived from primary tumor cell lines grown in hypoxic conditions, and from exosomes from plasma samples obtained from cancer patients. The SoRTEV™ RNA low volume kit was used according to its technical manual and the procedure presents two subsequent working steps: 1) exosome-associated RNA isolation from biofluid based on the incubation of immunobeads with the plasma sample and 2) column based RNA purification. The protein profile, size, EV type isolated (exosomes) have been previously reported in different solid tumors, including in a subpopulation of metastatic melanoma patients with Alix protein characterization as exosome marker using Western blot (Bernardi et al., 2019; Foroni et al., 2020; Zocco et al., 2020).

### 2.3 Measurement PD-L1, INF-γ, and TGF-β mRNA levels in EV

The mRNA levels of PD-L1, INF-γ, and TGF-β were measured using a QX200 digital droplet PCR (ddPCR, Bio-Rad, Hercules, CA), the One-Step RT ddPCR kit and the PrimePCR ddPCR Expression Probe Assay for CD274 (PD-L1, human, dHsaCPE5058502), INF-γ (human, dHsaCPE5034618), and TGF-β (human, dHsaCPE5055188). The human β-actin (ACTB, dHsaCPE5190200) ddPCR assay (BioRad, Hercules, CA) was used as a reference gene and internal control.

The One-Step RT ddPCR kit combine the first-strand cDNA synthesis (reverse transcription, RT) and qPCR reaction in one mixture. For each sample, 22 µL of One-Step ddPCR reaction was prepared using 1X as final concentration of each assay (FAM probe for target and HEX for reference gene, respectively) and adding 5 µL of RNA. Samples were partitioned into ∼ 20,000 uniform nanoliter-sized droplets in a 96-well PCR plate using the AutoDG Instrument (BioRad, Hercules, CA), with target and background RNA randomly distributed among the nanoliter-sized droplets.

The following conditions were used for the reverse transcriptase PCR reaction: 50°C × 60 min, 95°C × 10 min, 95°C × 30 s and 55°C × 60 s (40 cycles), 98°C × 10 min, 4°C hold.

mRNA expression was assessed by the automatic quantification of signals generated by the droplet reader using the QuantaSoft software (Bio-Rad, Hercules, CA). Data are reported as % fractional abundance (FA), calculated as follows: absolute quantification of mRNA copies of each biomarker (copies/mL)/absolute quantification of mRNA copies (copies/mL), including also β-actin (ACTB) as housekeeping gene. Each plasma sample was extracted once and ddPCR analyses were carried out in triplicate. Values are reported as a mean of the triplicate.

### 2.4 Computed tomography segmentation and extraction of radiomic features

Ten patients undergoing thoraco-abdominal computed tomography (CT) for disease staging who met homogeneity criteria for image acquisition parameters (Berenguer et al., 2018), were enrolled for radiomic analysis. Scan protocol homogeneity criteria included 120 kV tube voltage, 160 mAs exposure, a field of view between 36 and 40 cm, 1.5–2 mm slice thickness, and a standard/soft tissue convolution kernel. Only non-contrast CT images were used for radiomic analysis and CT scans were acquired at baseline. The radiomic analysis was performed by one author using the validated LifeX^®^ software (LifeX^®^, IMIV, CEA, Inserm, CNRS, Orsay, France) (Nioche et al., 2018), after appropriate manual segmentation of one hundred eighty-nine lesions (i.e., volumes of interest; VOIs). Thirty-six radiomic features including three shapes, two gray-level histograms, six gray-level co-occurrence matrices (GLCM), eleven gray-level run lengths matrix (GLRLM), three Neighborhood Grey-Level Different Matrix (NGLDM), and eleven Grey-Level Zone-Length Matrix (GLZLM) features, were computed (Supplementary Table S1).

### 2.5 Statistical analysis

Categorical variables, such as gender, Eastern Cooperative Oncology Group (ECOG) performance status, BRAF mutation, baseline lactate dehydrogenase levels, Neutrophil-Lymphocyte Ratio (NLR), the number of metastatic sites and the type of anti-PD1 were described by absolute and relative frequencies, while quantitative factor such as age at diagnosis was reported as median and range. The normal distribution of each variable was assessed by the Kolmogorov-Smirnov test.

In order to calculate the optimal cut-off value for mRNA levels of each gene, the Receiver Operating Characteristic (ROC) curve and the Youden’s Index analysis were applied. The relationship between the key genes and survival rate was analyzed using Kaplan–Meier survival curve analysis and the log-rank test was used to evaluate the differences between curves.

Progression-free survival (PFS) and overall survival (OS) were defined as the time from treatment start to disease progression or death. The Cox hazard regression method was used to identify independent risk factors for OS and PFS. At least 20 PFS events were expected to be sufficient to detect the treatment effect with 80% statistical power (5% type I error rate) with a hazard ratio (HR) set at 0.28. To overcome the problem of overfitting due to the small number of observed events (PD/death), the bootstrap technique was used to estimate the 95% CI for the hazard function and testing the assumption of the model. Furthermore, in order to calculate to which extent the radiomic features were correlated to the expression of immune-related cytokines in EVs, the least absolute shrinkage and selection operator (LASSO) logistic regression model adopting a 10-fold Monte Carlo cross-validation was used and executed in Matlab R2019a (MatLab^®^ software, The Math Works Inc., Natick, MA). LASSO is a method of regression analysis that improves the prediction accuracy by reducing the number of radiomic features and estimating the maximum-likelihood fitted regression coefficients for the remaining ones. Selected radiomic features for analysis are reported in Supplementary Table S1. Furthermore, the Spearman’s rank correlation analysis was conducted to test the relationship between radiomic features of melanoma lesions and the biomarker that was found to be predictive, while the nonparametric Mann–Whitney U test was carried out to assess differences in radiomic features to the optimal cut-off for the mRNA expression of the best predictive biomarker.

Differences were considered significant at *p* < 0.05. All statistical analyses were performed with MedCalc Statistical Software version 14.8.1 (MedCalc Software, Ostend, Belgium), the open-source statistical language R (R Foundation for Statistical Computing, Vienna, Austria) through the free and open statistical software program JAMOVI^®^ (Version 1.1.9; retrieved from https://www.jamovi.org) and SPSS version 26 (SPSS Inc. SPSS, Chicago, IL, United States).

## 3 Results

Thirty patients with metastatic melanoma treated with nivolumab (16 patients, 53.3%) or pembrolizumab (14 patients, 46.7%) monotherapy as first-line treatment were enrolled in this study. Clinical characteristics of patients are reported in Table 1.

**TABLE 1:** Clinical characteristics of patients.

	Patients (n = 30)
Age at diagnosis, median (years, range)	67.07 (62.39–71.73)
Gender	
***Male***	18 (60%)
***Female***	12 (40%)
ECOG PS	
***0***	19 (63.3%)
***1***	7 (23.3%)
***2***	4 (13.4%)
BRAF mutation	
***Yes***	7 (23%)
***No***	21 (70%)
***Unknown***	2 (6%)
Baseline LDH	
***Normal***	16 (53.3%)
***Elevated***	5 (16.7%)
*Missing*	9 (30%)
NLR score	
***<5***	21 (70%)
***≥ 5***	4 (13%)
***Missing***	5 (17%)
Stage AJCC VIII	
*M1a*	10 (33.4%)
*M1b*	4 (13.4%)
*M1c*	14 (46.6%)
*M1d*	2 (6.6%)
Metastatic sites	
***≤3***	18 (60%)
***>3***	12 (40%)
First-line treatment	
***Nivolumab***	16 (53.3%)
***Pembrolizumab***	14 (46.7%)

Abbreviations: American Joint Committee on Cancer (AJCC), Eastern Cooperative Oncology Group (ECOG) performance status, lactate dehydrogenase (LDH), Neutrophil-Lymphocyte Ratio (NLR).

Objective responses were 4 CR (13.3%), 7 PR (23.4%), 1 SD (3.3%) and 18 PD (60%).

The TGF-β mRNA levels expressed as fractional abundance (FA, %), were compared in both treatment groups. No statistical difference between the median TGF-β mRNA levels and the type of immunotherapy was found (*p* = 0.41).

**FIGURE 1:**
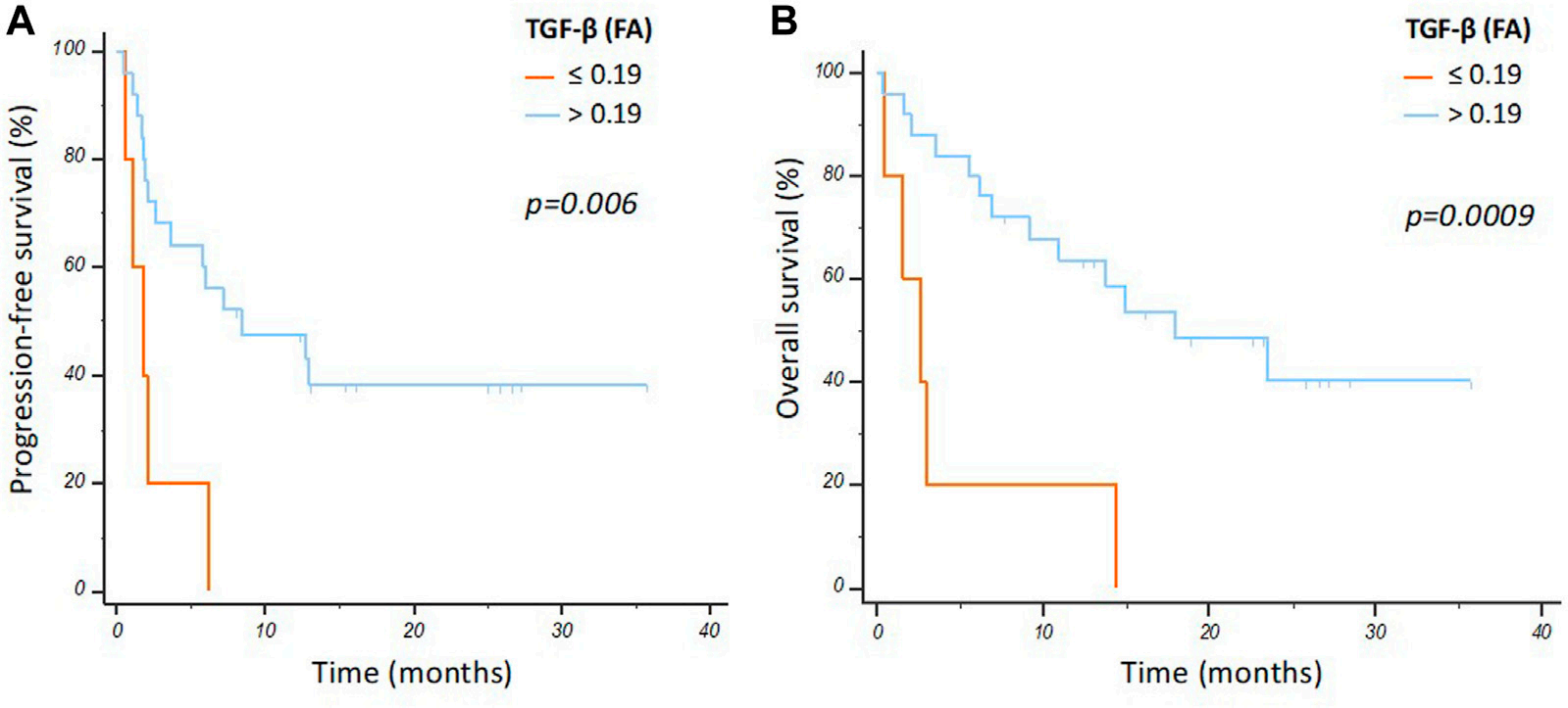
Progression free survival **(A)** and overall survival **(B)** curves according to TGF-β mRNA FA in the overall population.

When stratifying patients by optimal ROC-derived cut-off for TGF-β mRNA levels (FA, %), significantly longer median PFS (8.4 vs. 1.8 months; *p* = 0.006) and OS (17.9 vs. 2.63 months; *p* = 0.0009) were observed in patients with high vs. low TGF-β expression (FA > 0.19 vs. 0.19; Figures 1A,B).

Of note, the predictive value of TGF-β mRNA levels in EV was maintained, regardless of the anti-PD-1 agent used. Patients with high TGF-β mRNA levels given nivolumab showed a significantly longer PFS (8.4 vs. 0.5 months, HR 0.21) and OS (not reached vs. 0.5 months, HR 0.16), compared to those with low TGF-β expression (Supplementary Figures S1A, B). Similarly, patients treated with pembrolizumab with TGF-β mRNA levels above threshold (FA >0.19) showed a better PFS (5.8 vs. 2.1 months, *p* = 0.15; HR 0.39) and OS (17.9 vs. 2.9 months, *p* = 0.02; HR 0.25), compared to those with TGF-β FA ≤0.19 (Supplementary Figures S2A, B).

**FIGURE 2:**
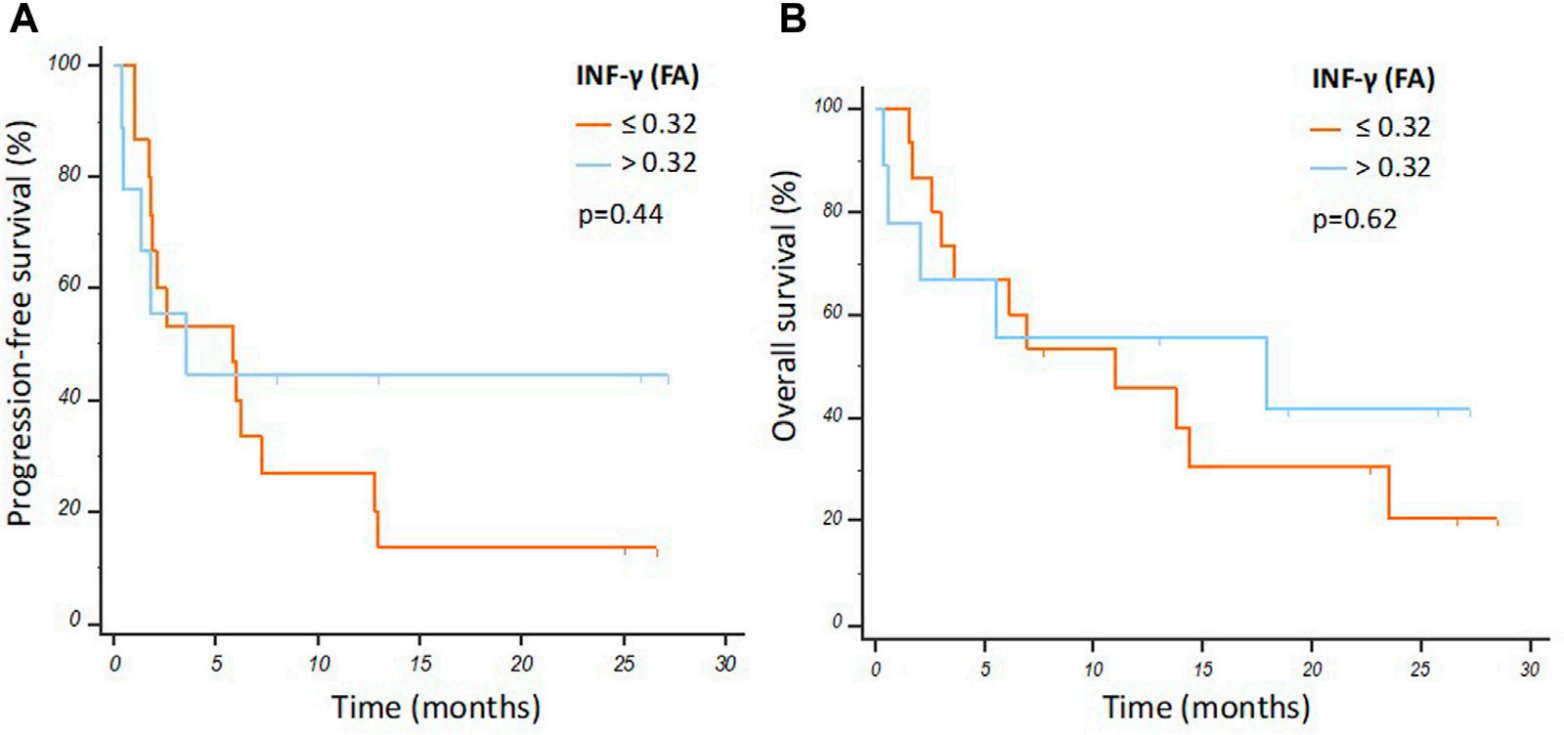
Progression free survival **(A)** and overall survival **(B)** Kaplan-Meier curves according to IFN-γ mRNA levels in the overall population.

No statistically significant differences were observed between INF-γ mRNA levels in EV and PFS or OS (Figure 2), while PD-L1 was undetectable in EVs.

The univariate analysis did not show significant correlation between clinical variables (age, gender, ECOG PS, anti-PD1 used, number of metastatic sites, NLR ratio, LDH, BRAF status) and PFS or OS (Table 2), confirming that TGF-β was the only independent biomarker associated with PFS and OS.

**TABLE 2:** Univariate analysis for PFS and OS.

	PFS		OS	
Variables	HR (95% CI)	*p*-value	HR (95% CI)	*p*-value
Age	1.01 (0.98–1.05)	0.47	1.01 (0.98–1.05)	0.39
Gender	1.98 (0.76–5.16)	0.16	2.38 (0.85–6.69)	0.10
ECOG PS	1.31 (0.38–4.46)	0.67	1.89 (0.54–6.57)	0.32
Anti PD-1	1.15 (0.48–2.77)	0.75	1.19 (0.47–3.01)	0.72
N. of metastatic sites	1.47 (0.59–3.60)	0.40	2.39 (0.91–6.24)	0.08
NLR ratio	1.77 (0.49–6.28)	0.38	3.04 (0.81–11.46)	0.10
LDH	2.92 (0.95–9.03)	0.06	2.83 (0.79–10.12)	0.11
BRAF mutation	0.65 (0.24–1.75)	0.39	0.63 (0.22–1.82)	0.39
TGF-β (FA)	0.25 (0.09–0.73)	**0.01**	0.19 (0.06–0.56)	**0.003**

Considering the relatively small number of samples included in the present study, a bootstrap analysis was performed to validate the effect of TGF-β on the prediction of time-to events outcomes. The PFS and OS univariate models (estimated from 1000-boot-strapped replications of the data) showed a strong significant association with the expression of TGF-β (*p* = 0.003 and *p* = 0.002) and a slightly significant association with LDH and NLR values (*p* = 0.04) (Supplementary Table S2). The multivariate analysis (estimated from 1000-boot-strapped replications of the data) did not show significant correlation between variables (LDH, NLR ratio, TGF-β) and PFS or OS.

**FIGURE 3:**
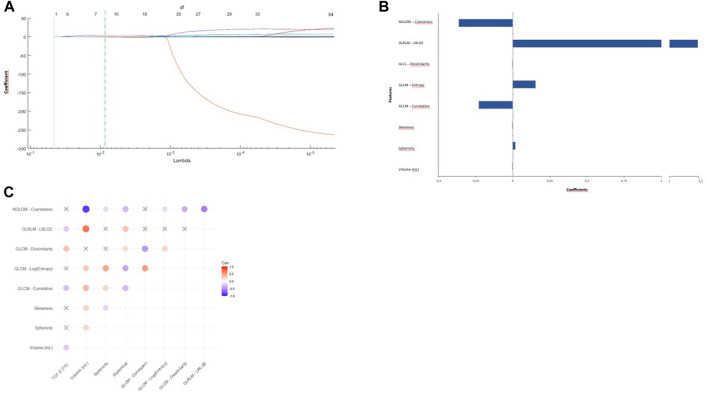
Radiomic features selection and their correlations. **(A)** Least absolute shrinkage and selection operator (LASSO) logistic regression model coefficient profiles (*y*-axis) of 36 radiomics features. The dashed vertical green line represents the optimal λ value (*x*-axis) which yield eight features with non-zero regression coefficients and minimal mean squared error, by performing 10-fold cross validation. **(B)** The *y*-axis indicates the selected eight radiomics features, and the *x*-axis represents the coefficient of the features. **(C)** Heatmaps describing Spearman’s correlation between the selected radiomic features and TGF-β mRNA levels in EVs. Bright red indicates direct correlation while blue the inverse one, with a gradient from red to blue indicating the Spearman’s correlation coefficient (r2). The larger the circle, the stronger the correlation. The crossed circles indicate non-significant correlations (*p* > 0.05). Abbreviations: FA, fractional abundance; mL, milliliters; Corr, correlation; p, *p*-value; r2, Spearman’s correlation coefficient; NGLDM, neighbor-hood grey-level different matrix; GLRLM, Gray Level Run Length Matrix; LRLGE, Long-Run Low Gray-Level Emphasis; GLCM, Gray Level Co-occurrence Matrix.

Then, radiomic features that passed the LASSO method, including NGLDM-Coarseness, GLRLM-LRLGE, GLCM-dissimilarity, GLCM-Log (entropy), GLCM-correlation, skewness, sphericity, and volume, were considered for the analysis of their correlation with TGF-β mRNA levels (Figures 3A,B). After robustness assessment, the correlation coefficient was calculated for each feature with respect to TGF-β mRNA levels in EV, and between each pair of features (Figure 3C, Supplementary Table S3).

**FIGURE 4:**
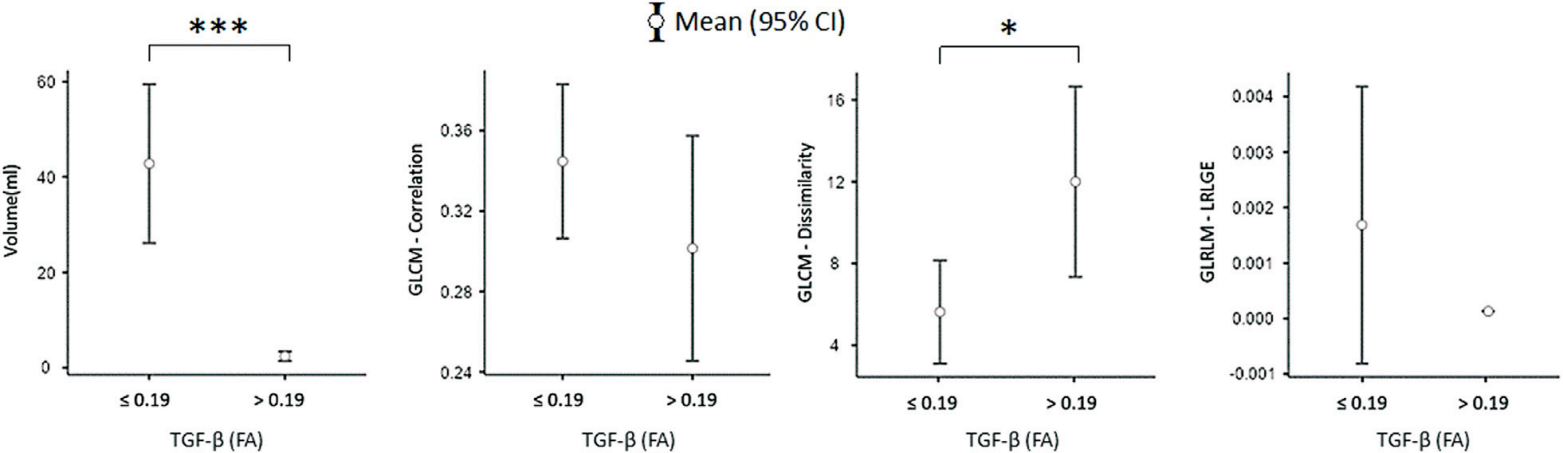
Comparison of radiomic features between patients with high vs. low TGF-β mRNA levels in EVs. *p < 0.05; ***p < 0.001. Abbreviation: FA, fractional abundance; mL, milliliters; GLCM, Gray Level Co-occurrence Matrix; GLRLM, Gray Level Run Length Matrix; LRLGE, Long-Run Low Gray-Level Emphasis; CI, confidence interval.

TGF-β at baseline was directly correlated with GLCM dissimilarity (r2 = 0.31, *p* < 0.001), while an inverse correlation was found with GLRLM-LRLGE (r2 = −0.22, *p* = 0.002), GLCM (r2 = −0.29, *p* < 0.001) and volume (r2 = −0.23, *p* = 0.001). Interestingly, patients with high TGF-β mRNA levels (FA >0.19) were found to have smaller lesions (2.41 ± 3.27 mL vs. 42.79 ± 101.08 mL, *p* < 0.001) (Figure 4) and higher dissimilarity (12.01 ± 28.23 vs. 5.65 ± 8.4, *p* = 0.018), as compared to those with low TGF-β FA (Figure 4).

Given the lack of PD-L1 expression at baseline and of predictive value of INF-γ mRNA levels, the correlation between these EV biomarkers and radiomic features was not investigated.

## 4 Discussion

Our findings demonstrated that high levels of TGF-β mRNA in circulating EVs were significantly related to longer PFS and OS in patients with metastatic melanoma treated with first-line nivolumab or pembrolizumab monotherapy.

In patients with solid tumors, including melanoma, elevated TGF-β plasma levels have been demonstrated to correlate with tumor progression, number of metastatic sites, and poor clinical outcome (Junker et al., 1996; Krasagakis et al., 1998; Wikstrom et al., 1998). Moreover, the predictive value of TGF-β signaling in anti-PD-1/PD-L1 treatments has been widely documented in solid tumours (Feun et al., 2019; Powles et al., 2019; Koh et al., 2020).

The novelty of the present study is based on the combination of the detection of TGF-β mRNA level using EVs, more representative of tumour heterogeneity, and radiomic analysis, which has the potential to be a clinically relevant readout of tumor microenvironment.

The feasibility of the reproducible workflow for the isolation of RNA from tumor EVs employing specific affinity-mediated selection beads allow its introduction in routine laboratory use (Bernardi et al., 2019; Foroni et al., 2020; Zocco et al., 2020). On the contrary, ultracentrifugation method, which is used for EVs isolation, requires a well-optimized protocol (no standardization is available), expensive instruments and time-consuming analysis procedures. Moreover, the results between studies are not comparable and there is a need for standardized methods as immune-affinity recognition kit.

Recent reports highlighted the high involvement of EVs in tumour proliferation, progression and therapeutic resistance (Konig et al., 2017; Dong et al., 2020; Kao and Papoutsakis, 2019; Fontana et al., 2021; Chen et al., 2022; Musi and Bongiovanni, 2023). Therefore, a “multiomic” strategy, combining liquid biopsy and radiomic data provides a better understanding of tumor molecular determinants and biological characteristics, providing a more efficient way to study predictive biomarkers of response to treatments using minimally invasive tools.

It has been recognized that tumors expressing high levels of TGF-β may be shielded from immune surveillance (Batlle and Massague, 2019). TGF-β inhibits the production of interleukin-2 (Kehrl et al., 1986) with suppression of T-lymphocyte differentiation and their effector function (Gorelik and Flavell, 2002; Yoshimura and Muto, 2011). Furthermore, TGF-β transported by melanoma-derived EVs inactivates antigen-presenting cells (Duchler et al., 2019).

Several lines of evidence showed that TGF-β can directly affect tumor development by inducing a dual effect. On one hand, TGF-β produced by tumor cells can induce antiproliferative effects on ectodermal-derived cells, including melanocytes (Batlle and Massague, 2019). On the other hand, when cancer cells lose TGF-β tumor-suppressive effects, they can use TGF-β signaling to promote differentiation into an invasive and metastatic phenotype (Batlle and Massague, 2019). Therefore, response of cancer to TGF-β may vary across different tissue types and disease stages (Tauriello et al., 2022). Busse and Keilholz reported increased levels of TGF-β in melanoma patients with disease progression, providing an optimal microenvironment for undisturbed tumor growth by promoting tumor cell motility, invasiveness and modulating tumor stroma and extracellular matrix (Busse and Keilholz, 2011).

Assuming that the TGF-β content in the EVs is related to its levels in the tumor, the main hypothesis to explain the association between high EV TGF-β mRNA levels and clinical response is that tumors overproducing TGF-β induce T-cell exhaustion that leads to decreased T-cell proliferation and function which may be successfully restored by the administration of anti-PD-1 antibodies.

We found that patients with high TGF-β mRNA levels in EVs had significantly smaller lesions than those with low TGF-β expression. This finding may be explained by the reduced T-cell recruitment in tumors due to the immunosuppressive effects of TGF-β. On the other hand, radiomic analysis showed that patients with high TGF-β mRNA levels also had high dissimilarity values, suggesting a greater tumor heterogeneity, which may have implications in immunotherapy response (Wu et al., 2019).

Radiomics is an emerging non-invasive analysis used to discover novel imaging biomarkers useful for patient classification and predicting treatment response. Several studies evaluated the performance of radiomic analysis in quantifying characteristics of tumor lesions, particularly in the context of immunotherapy (Trebeschi et al., 2019; Sun et al., 2022; Ungan et al., 2022). According to our data, it has been demonstrated a correlation between tumor heterogeneity identified using radiomics features and distinct immune responses patterns in triple-negative breast cancer patients (Jiang et al., 2022). Similarly, Henry et al. extracted radiomics features of tumors from CT scan of patients with metastatic lung cancer or metastatic melanoma, showing a significant inter- and intra-tumor heterogeneity (Henry et al., 2022). Interestingly, Grossman et al. evaluated the relationships between radiomic features, clinical factors, and tumor biology (i.e., immune response, inflammation) in lung cancer patients, demonstrating a correlation between radiomics and TGF-β receptor signaling (Grossmann et al., 2017).

These observations, together with the evidence showing that patients with a high TGF-β mRNA levels in EV could respond to anti-PD-1 antibodies regardless of the type of drug used, reinforces the hypothesis of a predictive role of TGF-β mRNA levels in patients with metastatic melanoma. Recently, De-Miguel Perez showed that high expression of TGF-β cytokine in EVs is associated with shorter PFS and OS in advanced non-small-cell lung cancer patients treated with ICIs (de Miguel-Perez et al., 2023). However, the EV TGF-β expression levels were evaluated by enzyme-linked immuno-sorbent assay, instead of TGF-β mRNA levels as in our melanoma cohort.

Nevertheless, PD-L1 mRNA was not detected in EVs in the present study. Several studies evaluated the expression of PD-L1 in melanoma (Kaunitz et al., 2017; Sunshine et al., 2017), particularly in EVs (Chen et al., 2018; Theodoraki et al., 2018; Cordonnier et al., 2020). Cordonnier et al. evaluated circulating exosomal-PD-L1 in melanoma patients by using an enzyme-linked immunosorbent assay, demonstrating a correlation with response to immunotherapy and clinical outcome (Cordonnier et al., 2020). Accordingly, Chen et al. reported that metastatic melanoma releases high levels of PD-L1 positive-EVs and that the level of circulating exosomal PD-L1 changes during the course of anti-PD-1 therapy (Chen et al., 2018). It should be noted, however, that these data were obtained with protein measurement, which may not be correlated with PD-L1 mRNA levels in EVs. Indeed, numerous molecular signals and transcriptional factors regulate PD-L1 expression such as the interferon regulatory factor-1 (IRF-1), which binds to the PD-L1 promoter (Garcia-Diaz et al., 2017; Theodoraki et al., 2018). IRF-1 is located upstream of PD-L1 in the IFN-γ-driven JAK/STAT signaling pathway (Lee et al., 2006) and has been shown to play a central role in regulating cancer cell responses to IFN-γ (Ascierto et al., 2011). Other transcriptional factors involved in PD-L1 regulation in melanoma include MYC, hypoxia-inducible factor-1α and 2α (HIF-1α/2α), STAT3 and NF-κB (Sun et al., 2018). Post-transcriptionally, PD-L1 expression can be negatively regulated by microRNA-34a (miR-34a) which induces PD-L1 mRNA degradation (Wang and Wang, 2017). Recently, Cortez et al. showed that elevated miR-34a expression induced by p53 transcriptional activity leads to a reduced PD-L1 expression in NSCLC (Cortez et al., 2016). Moreover, the authors analyzed NSCLC data from TCGA and reported significantly higher PD-L1 mRNA levels in TP53-mutated NSCLC than in wild-type samples. Thiem et al., 2019 analyzed skin cutaneous melanoma (SKCM) TCGA data and showed that PD-L1 mRNA levels were significantly higher in TP53-mutated melanoma samples (*p* = 0.0181). Therefore, the above-mentioned reasons could explain the undetectability of PD-L1 mRNA in our samples. Although the association between TGF-β and clinical response to immunotherapy is logical, a limitation of the present study is the small number of patients, the lack of a control cohort (i.e., metastatic melanoma patients treated with anti-BRAF agents) and of a comparison between TGF-β peripheral blood and tissue levels, which should be addressed in further studies enrolling a larger patient population.

## 5 Conclusion

The TGF-β mRNA profile in circulating EVs and the radiomic features could represent a clinically relevant readout of tumor microenvironment in patients with metastatic melanoma. Provided that the TGF-β mRNA levels in EVs may be associated with an increased expression of the growth factor into the tumor, the dual prognostic/predictive role of TGF-β may be the result of a combination of its tumor-suppressive/promoting effects. TGF-β mRNA in circulating EVs may represent a noninvasive biomarker in patients with metastatic melanoma treated with anti-PD-1 immunotherapy. However, we are aware that the present study reports data on a very small population of patients and further validations on larger cohort of patients are needed to confirm the biomarker role as predictive of immunotherapy response.

## Data Availability

The raw data supporting the conclusion of this article will be made available by the authors, without undue reservation.
