# Supplementary material for: TGF-β mRNA levels in circulating extracellular vesicles are associated with response to anti-PD1 treatment in metastatic melanoma
Source: Front Mol Biosci. 2024 Apr 3;11:1288677. doi: 10.3389/fmolb.2024.1288677 (PMC11021649; doi:10.3389/fmolb.2024.1288677)
Supplement: Supplementary file 1 [file DataSheet1.pdf]

Supplementary Material

TGF-β mRNA levels in circulating extracellular vesicles are associated with response to anti-PD1 treatment in metastatic melanoma

Stefania Crucitta[1§], Federico Cucchiara[1§], Riccardo Marconcini[2], Alessandra Bulleri[3], Simona Manacorda[2], Annalisa Capuano[4], Dania Cioni[3], Amedeo Nuzzo[2], Evert de Jonge[5], Ron H.J. Mathjissen[6], Emanuele Neri[3], Ron H.N. van Schaik[5], Stefano Fogli[1], Romano Danesi[1,7*], Marzia Del Re[1]

*** Correspondence:** Prof. Romano Danesi

Department of Oncology and Hemato-Oncology, University of Milano,

Via Festa del Perdono, 7, 20122 Milano,

Italy

Email: romano.danesi@unimi.it

1 Supplementary Figures and Tables

1.1 Supplementary Figures

Supplementary Figure 1. Progression free survival (PFS, A) and overall survival (OS, B) Kaplan Meier curves according to TGF-β mRNA levels in the nivolumab-treated population.

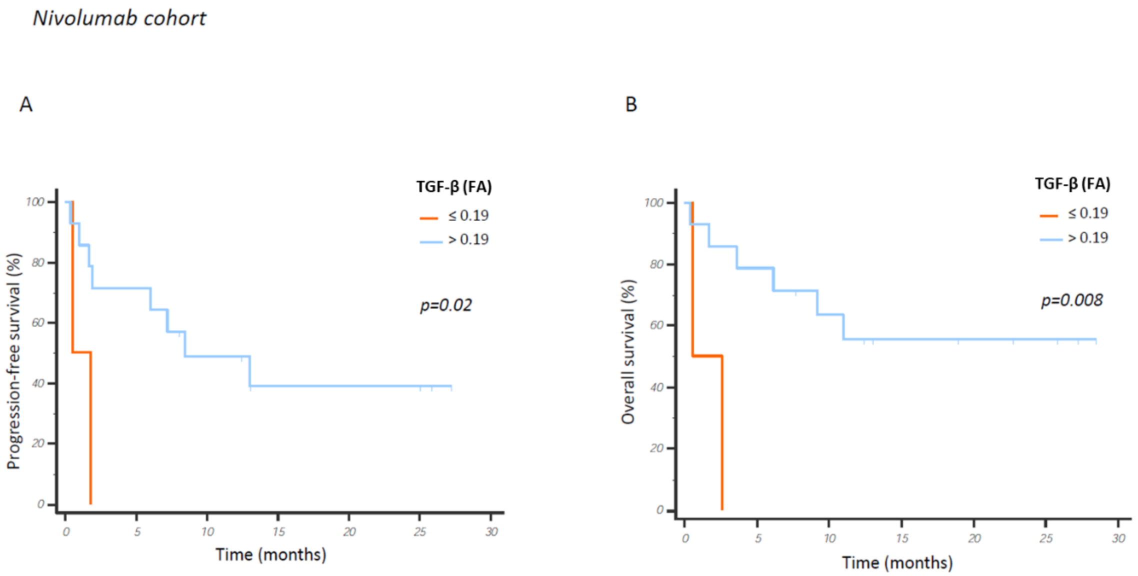

Supplementary Figure 2. Progression free survival (PFS, A) and overall survival (OS, B) Kaplan Meier curves according to TGF-β mRNA levels in the pembrolizumab-treated population.

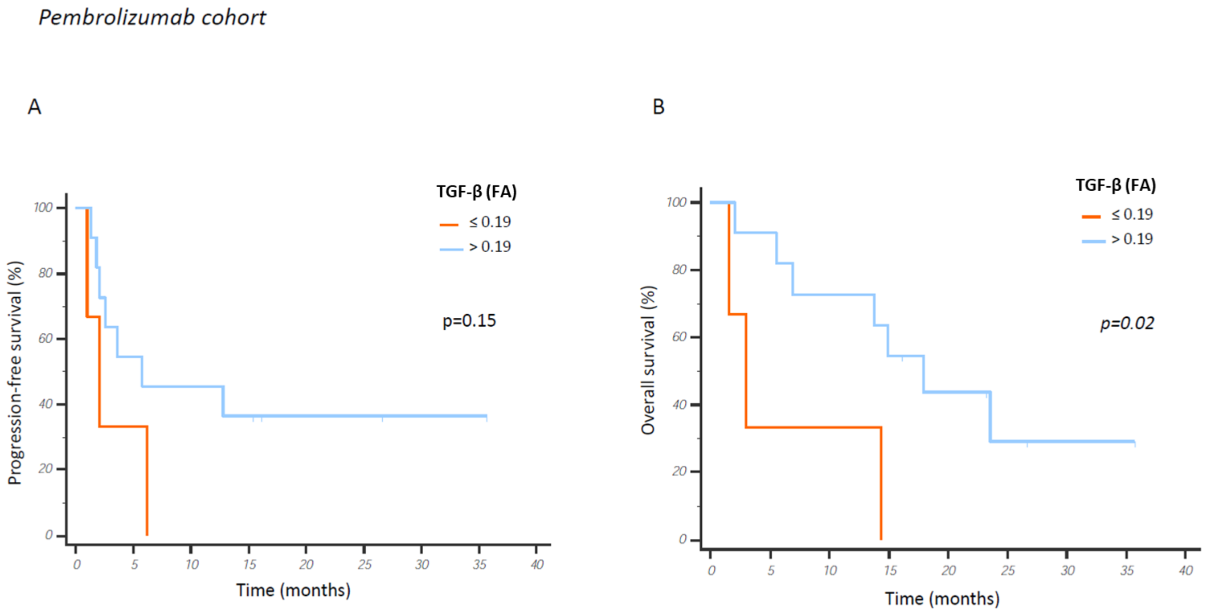

1.2 Supplementary Tables

Supplementary Table 1. Radiomic features evaluated in the study

**NGLDM - Coarseness**	Coarseness is a measure of average difference between the center voxel and its neighborhood and is an indication of the spatial rate of change. A higher value indicates a lower spatial change rate and a locally more uniform texture.
**GLRLM – Long-Run Low Gray-Level Emphasis (LRLGE)**	Long-Run Low Gray-Level Emphasis measures the joint distribution of long run lengths with lower gray-level values.
**GLCM – Dissimilarity**	Dissimilarity is another measure of heterogeneity that places higher weights on differing intensity grey-level pairs that deviate more from the mean
**GLCM – Log (Entropy)**	Entropy specifies the uncertainty/randomness in the image values. Entropy refers to tumor lesion heterogeneity
**GLCM – Correlation**	The correlation function evaluates the amount of regularity and the fineness/coarseness of the tumor texture. A higher value correlates with a greater irregularity in the neoplastic mass composition.
**Skewness**	Skewness refers to the Gaussian asymmetry that defines the histogram of the gray levels of the voxels defining the lesion. Skewness means lesion density.
**Sphericity**	Sphericity (= 1-eccentricity) is estimated from the ratio between the volume of the ellipsoid that could describe tumor and the volume of the spheroid. Sphericity (or better, eccentricity) describes how much tumor spread varies in space (i.e. whether or not it prefers one direction over the other). The lower the eccentricity, the greater the probability that the tumor grows in the 3 directions (x-y-z), rather than favouring one.
**Volume**	Lesion volume (ml)

Abbreviations: NGLDM: neighborhood gray-level difference matrix; GLRLM, Gray Level Run Length Matrix; GLCM, Gray Level Co-occurrence Matrix; ml, milliliters.

Supplementary Table 2. Univariate analysis for PFS and OS

	**PFS**		**OS**	
**Variables**	HR (95% CI)	p-value	HR (95% CI)	p-value
**Age**	1.01 (0.97 – 1.04)	0.41	1.01 (0.98 – 1.05)	0.28
**Gender**	1.98 (0.81 – 6.49)	0.14	2.38 (0.94 – 8.72)	0.67
**ECOG**	1.31 (0.04 – 21.37)	0.69	1.89 (0.05– 28.50)	0.38
**Anti PD-1**	1.15 (0.48 – 2.81)	0.74	1.19 (0.42 – 3.07)	0.70
**N° metastatic sites**	1.47 (0.61 – 3.77)	0.35	2.39 (0.89 – 9.08)	0.08
**NLR ratio**	1.77 (0.04 – 344.89)	0.31	3.04 (0.05 – 343.79)	***0.04***
**LDH**	2.92 (1.21 – 13.48)	***0.02***	2.83 (0.89 – 11.53)	***0.04***
**BRAF mutation**	0.65 (0.19 – 1.75)	0.40	0.63 (0.15 – 1.85)	0.43
**TGF-β (FA)**	0.25 (0.05 – 0.59)	***0.003***	0.19 (0.02 – 0.54)	***0.002***

Supplementary Table 3. Spearman Correlation Coefficient table for Inter-Relationships of radiomic features and TGF-β (FA). The 25 grey-shaded cells have statistically-significant associations, whereas the 11 clear cells have non-significant associations.

		**TGF-β (FA)**	**Volume (mL)**	**Sphericity**	**Skewness**	**GLCM – Correlation**	**GLCM – Log (Entropy)**	**GLCM – Dissimilarity**	**GLRLM – LRLGE**	**NGLDM - Coarseness**
**NGLDM - Coarseness**	r-value	0.06	*-0.85*	*-0.15*	*-0.31*	-0.11	*-0.15*	-0.35	*-0.56*	1.00
	p-value	0.43	*< 0.0001*	*0.04*	*< 0.0001*	0.13	*0.04*	*< 0.0001*	*< 0.0001*	
**GLRLM – LRLGE**	r-value	*-0.22*	0.68	0.004	*0.30*	0.12	-0.11	-0.08	1.00	
	p-value	*0.002*	*< 0.0001*	0.96	*< 0.0001*	0.11	0.13	0.26		
**GLCM – Dissimilarity**	r-value	*0.31*	-0.01	0.07	*0.17*	*-0.44*	*0.20*	1.00		
	p-value	*< 0.0001*	0.84	0.31	*0.01*	*< 0.0001*	*0.006*			
**GLCM – Log (Entropy)**	r-value	0.02	*0.27*	*0.44*	-0.40	0.49	1.00			
	p-value	0.75	*0.0001*	*<0.0001*	*< 0.0001*	*< 0.0001*				
**GLCM – Correlation**	r-value	*-0.29*	0.37	*0.22*	-0.31	1.00				
	p-value	*0.0001*	*< 0.0001*	*0.002*	*< 0.0001*					
**Skewness**	r-value	0.07	*0.22*	*-0.18*	1.00					
	p-value	0.31	*0.002*	*0.01*						
**Sphericity**	r-value	-0.03	*0.19*	1.00						
	p-value	0.71	*0.007*							
**Volume (mL)**	r-value	*-0.23*	1.00							
	p-value	*0.001*								

Abbreviations: NGLDM: neighborhood gray-level difference matrix; GLRLM, Gray Level Run Length Matrix; GLCM, Gray Level Co-occurrence Matrix; ml, milliliters.
